# Low-Energy Electron Damage to Condensed-Phase DNA and Its Constituents

**DOI:** 10.3390/ijms22157879

**Published:** 2021-07-23

**Authors:** Yingxia Gao, Yi Zheng, Léon Sanche

**Affiliations:** 1 State Key Laboratory of Photocatalysis on Energy and Environment, Fuzhou University, Fuzhou 350116, China; gyxmyr3719@163.com; 2 Département de Médecine Nucléaire et Radiobiologie et Centre de Recherche Clinique, Faculté de Médecine, Université de Sherbrooke, Sherbrooke, QC J1H 5N4, Canada; Leon.Sanche@usherbrooke.ca

**Keywords:** DNA damage, low-energy electrons, transient anions, dissociative electron attachment, radiotherapy

## Abstract

The complex physical and chemical reactions between the large number of low-energy (0–30 eV) electrons (LEEs) released by high energy radiation interacting with genetic material can lead to the formation of various DNA lesions such as crosslinks, single strand breaks, base modifications, and cleavage, as well as double strand breaks and other cluster damages. When crosslinks and cluster damages cannot be repaired by the cell, they can cause genetic loss of information, mutations, apoptosis, and promote genomic instability. Through the efforts of many research groups in the past two decades, the study of the interaction between LEEs and DNA under different experimental conditions has unveiled some of the main mechanisms responsible for these damages. In the present review, we focus on experimental investigations in the condensed phase that range from fundamental DNA constituents to oligonucleotides, synthetic duplex DNA, and bacterial (i.e., plasmid) DNA. These targets were irradiated either with LEEs from a monoenergetic-electron or photoelectron source, as sub-monolayer, monolayer, or multilayer films and within clusters or water solutions. Each type of experiment is briefly described, and the observed DNA damages are reported, along with the proposed mechanisms. Defining the role of LEEs within the sequence of events leading to radiobiological lesions contributes to our understanding of the action of radiation on living organisms, over a wide range of initial radiation energies. Applications of the interaction of LEEs with DNA to radiotherapy are briefly summarized.

## 1. Introduction

The damage induced to living organisms by high energy radiation (HER) is usually seen as occurring via a sequence of events, which can modify the irradiated cells and their behavior. These events can be broadly divided into physical, physico-chemical, chemical, and biological stages [1]. In the latter stage, the cell responds to the chemical transformations induced by the radiation. Except for neutron irradiation, during the physical stage, primary ionizations and excitations result from the propagation within biological tissue of charged energetic particles (i.e., fast electrons, protons, or ions) or fast electrons produced by primary high energy photons [2]. Most of the energy of these fast particles flows into the production of a large number (~4 × 10^4^ per MeV of deposited energy) of cations and secondary electrons (SEs). It is for this reason that SEs carry most of the energy deposited in cells by HER. SEs have energies ranging from zero to several hundreds of eV. The large majority produced below 20 eV is designated as low-energy electrons (LEEs). The most probable energy of SEs lies around 9–10 eV [3].

During the physico-chemical stage, cations and SEs recombine or react within femtoseconds, with molecules or subunits of large biomolecules in radiation tracks [4]. Both species produce excited states and radicals, but SEs can produce further ionization and transient anions (TAs). Dissociation of a TA result in the formation of a neutral radical and an anion (i.e., dissociative electron attachment; DEA) [5,6]. Alternatively, autodetachment of an extra electron from a TA can leave the molecule or molecular subunit in a vibrational or electronic excited state [7]. Dissociative electronically excited states resulting from both primary particle or SE interactions can lead to the creation of energetic ions and radicals [8,9,10]. When these species react with surrounding biomolecules before being thermalized; the phenomenon is referred to as “reactive scattering”. Both the latter type of reactions and electron-hole (cation) recombination are included in the physico-chemical stage [2,9,11]. Once the radicals, atoms, and new products induced by the radiation have thermalized, diffusion-controlled reactions occur as the irradiated system enters the chemical stage. At the end of this stage, the biological response is initiated, during which toxic products are eliminated and the damage is usually repaired. If not, mutation, necrosis or apoptosis can occur.

TAs are usually formed below 15 eV by capture of a SE into a previously empty orbital of a molecule or subunit of a large biomolecule. TAs have lifetimes varying from a few femtoseconds to picoseconds and can efficiently break chemical bonds via DEA, or by autoionization, when the neutral molecule or subunit is left in a dissociative excited state [7]. TAs constitute a major mechanism of LEE-induced damage. Since the pioneer research of Boudaiffa et al. [12], it has been amply demonstrated that when created within or close to DNA, LEEs and TAs can efficiently cause single strand breaks (SSBs), crosslinks (CLs), base modifications, double strand breaks (DSBs) and various other clustered lesions [6,13,14,15]. Unrepaired DNA clustered lesions in cells are responsible for mutagenic, genotoxic, and other potentially lethal effects [16]. LEEs are precursors of presolvated and solvated electrons, which also account for a portion of cellular radiation damage [11]. Consequently, defining the role of LEEs during the physico-chemical stage within the sequence of events leading to radiobiological lesions constitutes an essential element of our understanding of the action of HER on living organisms.

**Scheme 1:**
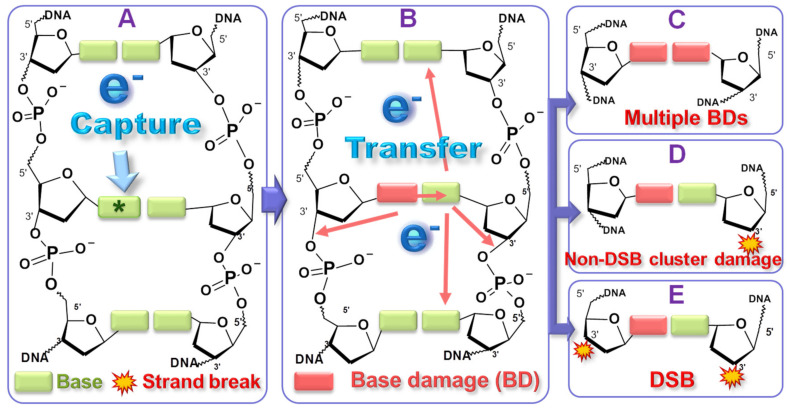
Diagram of the reaction pathways for a single LEE to produce all possible types of double-lesion cluster DNA damage. Possible electron transfer sites are indicated by red arrows. The electron (e-) is initially captured by a base, forming a core-excited transient anion (**A**). An electron autodetaches from the base anion, leaving the base in a dissociative state (**B**). The base is damaged and the detached electron transfers to other sites where DEA occurs (**B**). Transfer to the opposite base can result in two adjacent BDs (**C**), whereas transfer to the phosphate unit in the same or opposite strand, can cause a strand break via C−O bond breakage (SSB via DEA + BD) (**D**) or a DSB, if the BD on the left strand is converted to a strand break (**E**). Electron hopping between bases can create a BD or SSB farther away from the initial electron capture site (Copyright 2021 American Chemical Society [17]).

However, it has recently been shown that the initial capture of a single electron by a base, leading to TA formation within DNA, can directly cause potentially lethal cluster lesions, thus by-passing the chemical stage [17]. In other words, the well-established sequence of events is not necessarily followed in the case of TAs, since their decay followed by electron transfer within DNA can produce CLs and cluster lesions, as explained later in the text with Scheme 1. This is a single-hit mechanism that does not depend on the density of DNA damages, as in the case of cluster damages caused by multiple hits [18,19]. This TA mechanism does not rely on linear energy transfer (LET), as the ensuing cluster yields are linearly proportional to the number of LEEs along HER tracks [20]. When multiple events become effective in causing cluster lesions at high LET, the TA mechanism is still present, and its effects must be added to those created by multiple events [17]. The TA mechanism is different than the accepted mechanism leading to cluster lesions, which derives from random multiple hits by radiation-generated species along tracks that can cause two or more simple lesions, within one or two turns of the DNA helix [18,19,20,21]. Both a single LEE and multiple hits can produce a potentially lethal lesion within DNA during the initial (i.e., non-thermal) energy deposition process and hence bypass the chemical stage. These notions are particularly important to understand the radiobiological effectiveness of HER, which relates particle and photon irradiation to biological outcome [22]. Thus, understanding LEE-induced processes in DNA has implications, not only in the description of the physico-chemical stage of energy deposition, but also in explaining the production of DNA lesions potentially lethal to cells during the initial energy deposition process [17,23].

**Figure 1:**
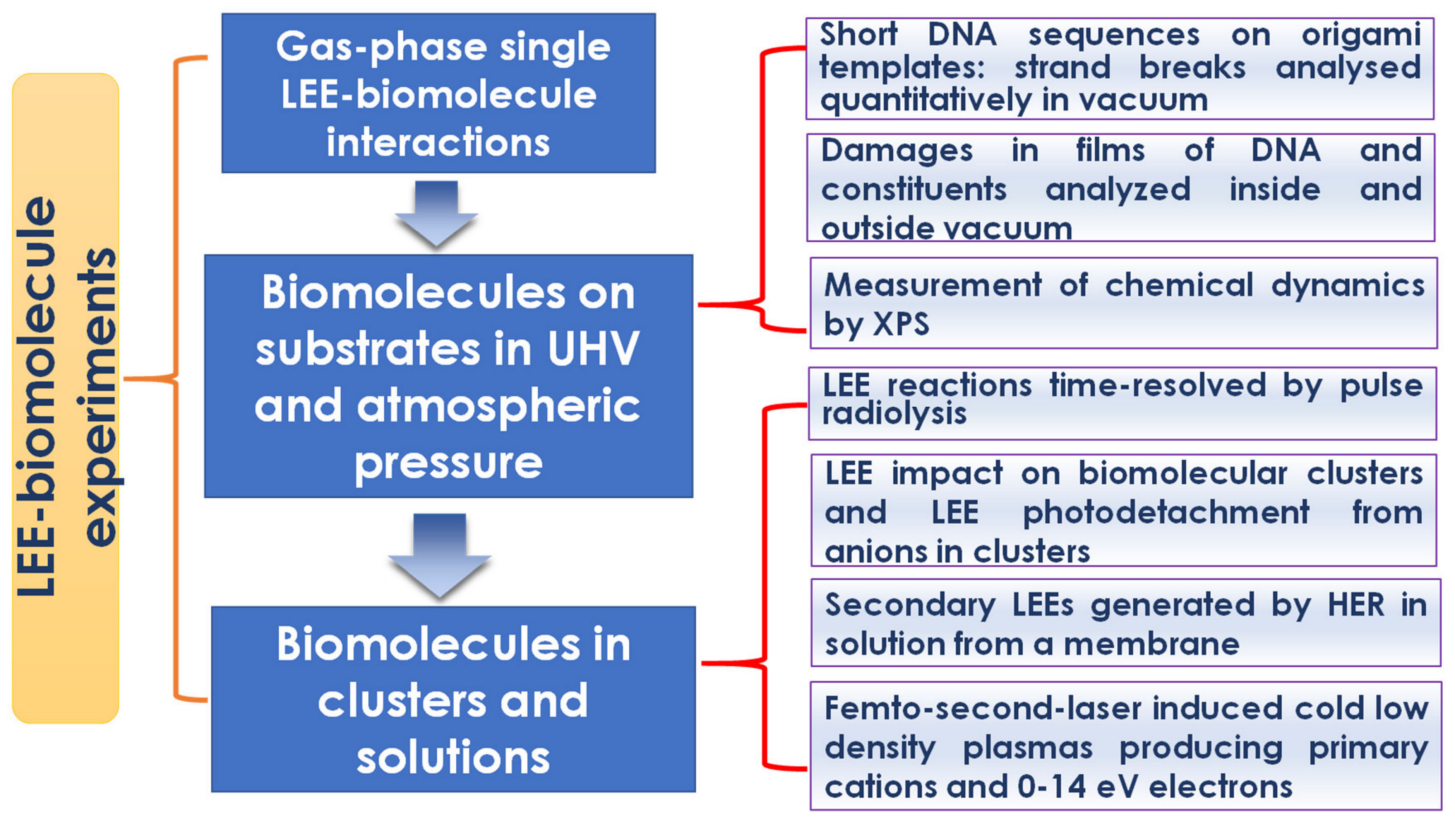
Scope of experimental investigations on the interaction of LEEs with biomolecules and DNA.

We first provide in the next section an outlook on the mechanisms of action of LEEs with molecules and their relationship to the damage they induce in DNA. Figure 1 gives an overview of the scope and status of the research on LEE interaction with biomolecules and DNA. It illustrates that basic knowledge from gas-phase electron-biomolecule experiments served to explain the behavior of LEEs in more complex systems and different types of condensed matter (e.g., clusters, solid films, water). Major contributions that helped transfer knowledge from the gas-phase to the condensed phase are briefly summarized in Section 3. The rest of the present review is limited to condensed phase results obtained from LEE experiments. It excludes results from radiation chemistry, including those obtained on the reactions of presolvated and solvated electrons with DNA and its constituents. In a review article, Sevilla et al. explain how some of the work and conclusions of the present article integrate into the ensemble of the chemistry triggered by HER in DNA [24].

As detailed in the right column of Figure 1, the reported results include those from (1) clusters of molecules or a specific biomolecule embedded within an aggregation of usually inert targets or water, (2) biomolecules condensed on substrates in ultra-high vacuum (UHV) and under standard ambient temperature and pressure (SATP), as well as (3) biomolecules in solutions. The molecules range from DNA fundamental constituents to condensed oligonucleotides, origami templates of short DNA sequences, self-assembled monolayer (SAM) of short DNA strands and bacterial (i.e., plasmid) DNA alone or mixed with other molecules in solid multilayer films. The present article is far from providing a complete description of all experiments and corresponding results that were published in this field, but informs the reader on significant contributions.

## 2. Mechanisms of Action of LEEs and Induced DNA Damage

The dynamics of LEE scattering in a dilute gas or within condensed material must be described in terms of wave functions. For a given situation and electron energy, the associated wave function can be expanded into a superposition of simpler plane waves. Intra- as well as inter-molecular interferences of such waves play a dominant role in LEE scattering within condensed matter. The scattering of LEEs from condensed atoms or molecule generates wave functions of wavelengths comparable to the size of molecules and the distances between them in biological media. In large biomolecules, LEE wavelengths are commensurate with the distances between the constituent fundamental units or molecular building blocks. Hence, intra- and inter-molecular coherent scattering modulate electron energy losses and molecular dissociations via localized (e.g., vibrational and electronic excitation) [25,26] and delocalized processes [27,28], including phonon [29,30] and exciton [31] creation and charge transfer [32,33]. Even in cells, where the random orientation of biomolecules and fast energy loss events can destroy long-range coherence, constructive interference of electron waves is still expected to persist, as shown in amorphous ice [34]. Furthermore, Caron and Sanche [35] demonstrated that the formation and decay of TAs are influenced by diffraction of LEEs within the DNA molecules. As a general rule, when disorder between condensed molecules increases, long-range coherent scattering of the electron waves arising from orderly positioned molecules diminishes and intra-molecular scattering phenomena become more dominant [36,37,38]. Hence, description of scattering processes in terms of the mechanisms of isolated electron–molecule interactions remain valid. However, these processes are modified by the presence of other neighboring targets and the band structure of the solid or liquid [5,35,39].

LEE scattering from isolated or condensed molecules can be considered as either direct or resonant [5,36,38]. Direct scattering occurs at all energies, because the interaction potential between the electron and the molecule is always present. The electron energy dependence of direct elastic and inelastic LEE scattering cross sections (CSs) exhibits a smooth, featureless, and usually rising signal. In contrast, resonance scattering occurs when the target atom or molecule temporarily captures the incoming electron into an additional orbital [5,37,39]. The resulting TA lies at the energy of this orbital [5,13,39]. At this energy, the inelastic scattering intensity and/or product yields are enhanced, exhibiting a pronounced maximum usually superimposed on an increasing background signal arising from direct scattering.

The formation of TAs and their decay channels have been amply described and reviewed in the literature [5,6,11,13,14,37]. The perturbating effects of water on TA formation and decay has also been reviewed recently with emphasis on theoretical progress [40]. A TA state has an intrinsic width in energy that depends on its lifetime. The resonance width can be determined from the dependence on electron energy of elastic scattering, a particular energy-loss process or product yields. There are two major types of TAs [5,13]: shape and core-excited resonances. Single-particle or shape resonances occur when the attaching electron occupies an otherwise unfilled orbital of the target molecule in its ground state. Such an electron capture can also occur at the site of a fundamental or subunit of a large biomolecule, such as DNA [13,41]. Core-excited resonances or ‘two-particle, one-hole’ states result from electron capture by the positive electron affinity of an excited state of the target molecule, or fundamental subunit of a large biomolecule, such as a base, sugar, or phosphate group in the DNA molecule. A core-excited anion can re-emit the electron (i.e., autoionization) into the scattering continuum or another site of a large molecule [39], leaving the target in the ground state or in excited rotational, intramolecular, and intermolecular (i.e., phonon) vibrational modes, as well as in electronically excited states. Dipole-bound electron resonances constitute another category of TAs that can lead to fragmentation or electron stabilization [42,43,44,45]. In this case, beyond a critical value of the dipole moment, the electron is captured by the long-range dipole potential of a target molecule. However, the electron-dipole interaction extends beyond the distances between molecules in condensed matter [42,43]. Dipole-bound anion are therefore not expected to exist in that phase, because of the overlap of the electron-molecule potentials of neighboring molecules [35]. In the case of water clusters for example, up to about 10–12 water molecules, the excess electron remains in the dipole potential [44]. Otherwise, water molecules form one or more complete hydration shells, causing the additional electron to migrate into an orbital localized on the outer surface of the water cage [44]. Related results are mentioned in Section 6 of the present article.

Repulsive TA states can dissociate into a neutral radical and a stable anion (i.e., DEA), but when the lifetime is too short, autoionization occurs. The measured width of a DEA peak in the anion signal results from the convolution of the intrinsic width and the line shape of projection of the ground state nuclear wavefunction onto the anion’s dissociative potential-energy curve in the Franck-Condon region. For DEA to occur the anion’s lifetime must be of the order or greater than about half a vibration period along the corresponding dissociation coordinate [33]. In other words, if the lifetime is too short the additional electron leaves the molecule before dissociation of the TA. Electron autodetachment considerably increases the vibrational excitation CSs of the target molecule, producing broad peaks in their energy dependence [7]. Competition between dissociation and autoionization of a TA is very sensitive to the environment, since both depend exponentially on the lifetime of the TA [5,38]. In turn, this lifetime is highly sensitive to the band structure and the new decay channels of the condensed medium, as well as perturbations of the electron-molecule or electron-subunit potential arising from neighboring molecules [38,39,46].

Below about 15 eV, electrons can temporarily attach at specific energies to DNA and its fundamental subunits [41,47]. In short oligonucleotides, the incoming LEE can be captured, with similar probabilities, by the phosphate group or by a base [41,47]. In long duplex DNA or plasmids, electrons with energies below 15 eV are mostly captured by a base, forming a TA of that base [15,48]. Below 4 eV, only shape resonances can locate an electron in an otherwise unfilled orbital of a base since electronic excitation is not energetically possible. Martin et al. reported the presence of two maxima at 0.8 and 2.2 eV in the electron-energy dependence of the yields (i.e., the yield function) of SSBs in plasmid DNA [49]. These enhancements were interpreted as shape resonances, resulting from electron attachment into the otherwise empty π* valence molecular orbitals of the DNA bases [50]. Dissociation of the base TA can produce an abasic site or a base damage (BD) [15,47]. As predicted theoretically, SSBs can also occur due to electron transfer from this π* orbital to a low-lying σ* orbital of the phosphate group, forming a dissociative TA at that site [41,50]. At higher energies up to about 15 eV, this type of process occurs via core-excited resonances, from which an electron can also transfer to the sugar-phosphate group [15,41,51,52]. Thus, over the entire 0–15 eV range, a TA formed on a base can dissociate via DEA producing an abasic site or a BD [15]. Moreover, an electron can autodetach from a base TA, while leaving the base in a dissociative electronic excited state. The detaching electron can also transfer to another fundamental unit, where DEA can occur [15,41,48]. *Thus, simultaneous base dissociation and electron transfer within DNA open channels for multiple damages induced by a single electron.* The possibilities are explained schematically in Scheme 1.

Frame A on the left shows an electron of energy larger than 4 eV being captured by a base to form a core-excited TA. In the middle frame (B), an electron from the TA detaches from the base, while the latter is damaged from being left in a dissociative electronic excited state. The autodetaching electron can transfer to a nearby site or more distant site in the same or opposite strand, where another TA is formed and decays via DEA. Depending on the site of attachment of the transferred electron (i.e., a base or phosphate group), three different types of local cluster damage can be induced, as shown in the frames on the right. They include (C) double BD and (D) a strand break (SB) with a nearby BD. If BDs in C or D are converted to SBs, then a DSB is formed (E). *Thus, by this mechanism DNA cluster lesions that are potentially lethal to cells can be induced within femtoseconds following initial ionization by HER.*

## 3. Transfer of Knowledge from the Gas to the Condensed Phase

During the past two decades, the consequences of LEE-biomolecule and LEE-DNA interactions were probed under different experimental conditions. With the aid of theoretical calculations and models [35,50,53,54,55], it has been possible to establish, the major mechanisms responsible for damaging DNA, a biomolecule that is central to cell function and the main target of radiotherapy [19,22,56,57]. Many authors have shown that the basic principles of isolated LEE-molecule scattering and LEE-induced processes, known to occur in simple molecules in the gas-phase, could be applied to the more complex biomolecules including DNA and its subunits [6,39,58]. A multitude of experiments on LEE-impact on thin films of condensed simple organic and biological (e.g., O_2_ and H_2_O) molecules paved the way to our present comprehension of LEE-DNA interactions [37,59,60,61]. Illenberger and his group have been particularly effective in linking electron processes in the gas phase to those in the condensed phase [62,63,64,65]. Their laboratory systematically investigated LEE-induced processes in isolated, clustered, and condensed molecules. By crossing an electron beam with a jet of molecules in vacuum, they studied the formation of TAs and DEA by anion mass spectrometry. In such electron-molecule crossed beam experiments, they investigated DEA from gaseous simple organic molecules (e.g., halomethanes, ethanol, trifluoroethanol) [62,63,64,65,66] to the DNA bases thymine and cytosine [67] and the DNA backbone using deoxyribose-ring model compounds [68]. Although this review is limited to condensed-phase data, it is worth mentioning that their results on gaseous C_4_H_4_O, C_4_H_8_O, and C_6_H_12_O_6_ supported the concept that in DNA the sugar moiety takes an active part in the initial molecular processes leading to SSBs [68]. Alternatively, Konig et al. simulated the behavior of LEEs attacking the phosphate group in DNA with dibutyl phosphate as a model compound [69]. They suggested that the most direct mechanism leading to SSBs at sub-excitation energy (<4 eV) should be DEA to the phosphate group, leading to scission of the C-O and P-O bonds [69]. Furthermore, Bald et al. were able to vaporize D-ribose-5-phosphate through laser-induced desorption, showing that near 0 eV electrons could be resonantly captured by this model compound of the DNA and RNA backbone, which subsequently led to cleavage of the sugar–phosphate bond [70]. These findings supported the idea of strand cleavage via direct DEA to the DNA/RNA backbone.

In a multitude of related gas-phase and cluster experiments, the group of Scheier was also involved in demonstrating the applicability of fundamental LEE-interaction mechanisms to biomolecular damage [68,71,72]. In particular, they demonstrated that below 3 eV, the DEA energies of the nucleobases and analog compounds were sensitive to the site of electron attachment within the nucleobases [70,73]. In other words, an electron of a specific energy can cleave a specific bond, even in a complex biomolecule. For further information on these topics the reader is referred to previous review articles [6,58,64,74].

## 4. LEE Impact on Biomolecular Films in UHV

### 4.1. Damage Analysed in Vacuo

Multi- or sub-monolayer films of a biomolecule deposited on a conductive substrate, or an inert spacer layer deposited on a metal substrate, have served as condensed-phase targets. Analysis in vacuum has been mainly performed with oligonucleotides or smaller fundamental DNA units. Condensed-phase electron energy-loss spectra for LEE scattering from fundamental DNA constituents (e.g., phosphate, sugar, bases, and nucleosides) and some of their analogs have been investigated for the last two decades. Since two review articles [7,75] have recently appeared on absolute electron-scattering CSs derived from high-resolution energy-loss spectra, this subject is not further discussed in the present article.

Some of the damage induced by LEE impact on thin multilayer films of various types of DNA and its constituents were analyzed in UHV by electron stimulated desorption (ESD) of stable anions and neutrals. These species were formed by DEA, dipolar dissociation, and dissociative electronic excited states. Target molecules included deoxyribose analogues [76,77], bases [78,79], and halouracils [80], the phosphate group [81], oligonucleotides, halogenated oligonucleotides [82,83,84,85,86,87,88,89], and duplex DNA [51,52]. The anions H^-^, O^-^, OH^-^, CN^-^, and OCN^-^ produced by DEA could be correlated to the fragmentation of specific bonds or components. The effect of hydration [90], oxygen [91,92], and morphology [93] were investigated. The most significant findings are summarized below.

By orienting 40-mer oligonucleotides as SAM perpendicular or parallel to a gold substrate, Pan and Sanche determined the orientation dependence on the site of LEE attack and the corresponding mechanism of damage [52]. Formation of OH^-^ could be attributed to DEA to the phosphate group, when it contains the counterion H^+^. Ptasinska and Sanche [88] measured the ESD of H^-^, O^-^, OH^-^, CN^-^ and CNO^-^ from the short oligonucleotide GCAT and compared their results to those obtained with fundamental units in the gas phase and longer condensed-phase duplex DNA. The TAs below 12 eV decayed via single or complex multi-bond dissociation to a stable anion and a neutral fragment. They concluded that in the longer double-stranded DNA (i.e., 40 and 3197 base pairs), the DEA process should not be considerably influenced by long-range electron interactions. Further experiments with abasic GCAT indicated that in addition to the absence of electron capture by the missing base, electron coherence is reduced within the oligonucleotide. This reduction and the lower capture efficiency were expected to decrease bond scission via DEA [89].

Mirsaleh-Kohan et al. [91] measured the desorption of anions stimulated by 1–18 eV electron impact on SAMs of single DNA strands of 10 nucleotides as a function of film temperature (50–250 K). When SAMs that had been pre-exposed to electrons were dosed with O_2_ at 50 K, the O^-^ and OH^-^ signals increased. The latter signal was further enhanced upon heating, while that of O^-^ decreased. This behavior was attributed to reactions of O_2_ with DNA damage sites and, upon heating, to thermally activated reactions between COO radicals and DNA to yield COH groups. These results were consistent with the “oxygen fixation” effect well-known in radiobiology [94]. When a film of GCAT was covered by 3 monolayers (MLs) of water, corresponding to 5.25 water molecules per nucleotide, the desorption of H^-^, O^-^, OH^-^ induced by 5–20 eV electrons resulted principally from a new type of dissociative core-excited TAs formed via electron capture by a DNA-H_2_O complex [90]. The overall anion yields emanating from the mixed-layer film increased by a factor of 1.6 with adsorbed water. O^-^ ESD via DEA from highly uniform thin films of DNA molecules intercalated with diaminopropane was investigated by Boulanouar et al. [95]. The molecular arrangement mimics the binding of proteins to DNA. Compared to “pure” DNA films, O^-^ ESD yields decreased, while OH^-^ increased due to the binding of diaminopropane to phosphate groups. Generally, the ESD results demonstrated that the type and yields of DNA damages induced by LEEs could be greatly affected by the environment, orientation of the molecule, base identity, and sequence.

Experiments with short DNA sequences assembled on a DNA origami template deposited on a semiconductor substrate were introduced by the group of Bald [96]. With the binding of phosphorescent Streptavidin to biotinylated oligonucleotide origami anchored on Si/SiO_2_ substrates, the new strategy could visualize the electron-induced dissociation of a single chemical bond, i.e., a SB, [97]. Atomic force microscopy was employed to image and quantify LEE-induced bond dissociations within these specifically designed single-strand DNA targets. The technique enabled the immediate determination of DNA SB yields in vacuum, with unprecedented control, over the DNA’s primary and secondary structures, i.e., nucleobase sequence and hybridization state [96,97].

The CSs and quantum yields of SBs in four different 12-mer oligonucleotides induced by ultraviolet (UV) photons and 6.5–9 eV electrons were measured by Vogel et al. [98]. Under identical conditions, the same targets deposited on Si substrates were irradiated with 8.44 eV vacuum ultraviolet (VUV) photons and 8.8 eV electrons. The SB CSs for LEE irradiation at 8.8 eV were found to be one to two orders of magnitude larger than the ones obtained with VUV photons; only a slight dependence on base sequence was observed. DEA was the dominant dissociative pathway leading to the considerably higher SSB CSs. The results help to assess and compare the DNA damage induced by photons and electrons close to the ionization threshold [99]. Recently, variations of the yields of strand scission on oligonucleotide length and energy in LEE-irradiated origami templates were investigated systematically [100]. The CSs for these yields was found to depend on both parameters, with the largest increase in the yields occurring at 7.0 and 8.4 eV. The DNA origami technique showed considerable potential for the development of brighter and more sensitive reporters for fluorescence-based detection schemes, such as microbead-based assays [101]. Such methods are useful in the development of biomarkers for early diagnosis of diseases in diagnostic applications [102]

Details of the mechanism of action halogen radiosensitizers were provided by the group of Bald, from further experiments with the origami technique. They investigated LEE-induced SBs in short DNA sequences that were modified by halogenated nucleosides [103,104]. Substituting the radiosensitizers BrU and FU for thymine and FA and BrA for adenine into the sequence resulted in the increase of the SB CSs [103,104]. The studies indicated that if halogenated radiosensitizers target the right nucleotide sequences in a tumor, the larger damage CSs could lead to more effective radiotherapy.

The group of Naaman measured the LEE (<2 eV) current transmitted through SAMs of short DNA oligomers to estimate the electron-capture efficiency [105]. For single-stranded oligomers the capture probability scaled with the number of guanine bases and was dependent on their clustering level. Double-stranded DNA did not capture electrons as efficiently as single-stranded oligomers, but once captured, electrons were more strongly bound than in single strands. By applying wavelength dependent low-energy photoelectron transmission and two-photon photoemission spectroscopy, Markus et al. investigated the density of states of DNA oligomers with partial sequence elements of the human telomere as SAMs on gold [106]. These experiments provided further evidence of electron capture by fundamental DNA units (i.e., formation of TAs) and their dependence on base sequence. Furthermore, this group compared electron capture by DNA and peptide nucleic acid (PNA) strands [107]. PNA is an analogue of DNA, which has a pseudopeptide backbone. In both compounds, guanine could capture electrons more efficiently than thymine. The electron stabilization process is different for the two molecules: in PNA the electron locates on the base and the structural changes occur to stabilize the electrons, whereas in DNA, after being captured by the base, the electron transfers efficiently to the backbone.

The magnitude of the current of electrons transmitted through film of chiral molecules depends on electron spin orientation. This chirality-induced spin selectivity (CISS) effect [108] was investigated in double-stranded oligomers in the experiments of Xie et al., who found that DNA can act as a spin filter for LEEs [109]. From such experiments, Kumar et al. developed a device that allows direct measurement of spin selectivity in charge transfer processes occurring in adsorbed molecules [110]. In principle, this selectivity can operate in UHV as well as in solutions. The technique was latter applied to other systems [111,112]. Knowledge from the variety of experiments performed in Naaman’s group led to identification of optimal conditions for the self-assembly of single- and double-stranded DNA on silver films. The results enabled the efficient use of silver substrates in plasmonic-based sensors of DNA and other biological applications [113].

X-ray photoelectron spectroscopy (XPS) also served to probe specific damages in thin films of DNA and constituents [114]. The technique helped exploring the chemical dynamics of damage mechanisms under UHV. Prior to analysis by XPS, the target film supported by a conductive substrate was either irradiated by an electron gun or by photoelectrons produced by the incident X-rays. The first experiments of this type with DNA components and related radiosensitizers were performed over two decades ago by Klyachko et al., who investigated the properties of halouracils (5-FU, 5-BrU, and 5-IU) [115]. They showed that both SEs from 1.5 keV X-ray irradiation of their MoS crystal substrate and near 0 eV electrons from a gun induce dissociation of 5-halouracils into a halogen anion and a uracilyl radical. The high anion yields in 5-BrU and 5-IU films were explained by the enormous CS for DEA of near 0 eV electrons. *When these halogens were imbedded in a frozen water film the halogen anion yields increased by at least an order of magnitude.* The smaller yields of F^-^ were due to the higher-energy threshold for DEA to 5-FU, which lies near 2 eV, and therefore prohibits dissociation by near 0 eV electrons. These authors also performed similar experiments with the four DNA bases [116].

Using X-rays (950 eV) as a probe and source of secondary LEEs (<20 eV), McKee et al. measured the radiation damage to the nucleotides 2′-deoxyadenosine 5′-monophosphate (dAMP), adenosine 5′-monophosphate (rAMP), 2′-deoxycytidine 5′-monophosphate (dCMP), and cytidine 5′-monophosphate (rCMP) deposited on a gold surface [114]. The X-ray fluence behavior of O-1s, C-1s, and N-1s photoelectron transitions was analyzed to obtain the phosphate, sugar, and nucleobase damage CSs. The phosphate cleavage CSs for dAMP, rAMP, dCMP, and rCMP were 23.4, 22.3, 30.7, and 32.8 Mb (1 Mb = 10^−18^ cm^2^), respectively [114]. The CSs of sugar lesions between dAMP, rAMP, dCMP, and rCMP were statistically similar to those of the phosphate, while nucleobase damage was relatively uniform with a low CS (~5 Mb) [114]. McKee et al. speculated that the observed damage was mainly inelastic energy loss channels related to SE capture and TA decay. They proposed that autoionization-mediated decay of TAs produced lower-energy electrons and that the shape resonances formed by capturing these electrons lead to bond breakage within a local scattering region with high efficacy [114]. Any radicals produced by the released electrons or DEA, further enhanced the density of damage at very low electron energies. Recently, Kundu et al. analyzed chemical modifications in condensed multilayer film of dAMP after direct electron irradiation in the range 3–25 eV by XPS [117]. These authors also remarked that in the energy degradation process of SEs, most of them end up at very low energies (i.e., 3 eV and below); consequently, they stressed the importance of the low-energy shape resonances [49] in DNA radiation damage [117].

Surface-enhanced Raman microscopy of graphene was introduced by Sidorov and Orlando, as a unique platform for monitoring and measuring LEE-induced DNA damage [118]. The graphene samples were synthesized by chemical vapor deposition on polycrystalline Cu foils and transferred onto gold covering a Si/SiO_2_ substrate. The latter was covered with single stranded DNA and bombarded with ≤1 eV electrons. The p-graphene on Au enhanced DNA breakage due to ballistic-electron transfer access to the dissociative sugar-phosphate shape resonance. The technique allowed direct and rapid assessment of LEE-induced damage via nucleobase electron capture and electron transfer to the C−O−P σ* extra-electron orbitals [41,52,118].

### 4.2. Damage Analysed Quantitatively Ex-Vacuo

Due to the complexity of the analysis, all products induced by LEEs on an entire DNA plasmid cannot be determined without digesting the molecule, in which case some of the details are lost. Compared to large biological DNA, constituents such as oligonucleotides are therefore better targets to elucidate the chemical reaction pathways induced by LEEs in UHV. However, for chemical analysis by high-performance liquid chromatography (HPLC) (combined or not with MS) of the details of the reactions induced in these DNA strands, the currents emitted from high spatial and energy resolution electron guns are too low and the targets too small to produce sufficient degraded material. A special apparatus has been constructed to alleviate these problems [119]. With this apparatus, many of the products induced by non-ionizing or weakly ionizing electrons, in the range 0.1 to 12 eV, incident on short chains of the DNA molecule have been analyzed [41,47,120,121,122,123,124,125,126,127,128,129,130].

In such experiments, oligonucleotides were spin coated on the inside of a Ta cylinder to form a uniform film. LEE bombardment of such films produced a sufficient amount of products for chemical analysis [119,120,121,122,123,124]. Afterwards, the molecules were dissolved in methanol and the products analyzed and quantified ex-vacuo by HPLC-UV or HPLC/MS-MS. The results obtained with short thymidine oligonucleotides, including monomers to tetramers (CGTA, GCAT), pT, Tp, pTp, TpT, pTpT, TpTp, pTpTp, TpTpT, TXT (X represents one of DNA bases A, G, C, T), and brominated oligonucleotide trimers (TBrXT) [47,120,121,122,123,124,125,126,127,128,129,130] indicated that (1) temporary LEE attachment a nucleoside primarily lead to *N*-glycosidic bond cleavages, liberating nonmodified bases [120,129]; (2) when the electron is captured directly by a phosphate group at the end of the strand, the total damage increases, but the release of bases is diminished [47]; (3) when the electron resides inside the chain on a phosphate strand scission occurs with a higher probability. Similar reactions with tetramers were found to involve DEA leading to phosphodiester C-O bond cleavage via electron transfer from the bases [121]. Damage was also affected by base sequence and bromination [123,124,127]. The presence of amino acids influenced the DNA damages induced by LEEs [131]. Glycine at low ratios with GCAT increases the total fragmentation yield induced by 1 eV electron, while arginine had a protective effect.

The release of non-modified thymine base from thymidine upon exposure to monoenergetic 10 eV electrons, which implicates cleavage of the C-N glycosidic bond, was first demonstrated by Zheng et al. [120]. In tetranucleotides, in addition to base release, the formation of fragments with a terminal phosphate was induced by 10-eV electron impact [121]. These fragments pointed to cleavage of the C-O bond of the phosphodiester group, a reaction that leads to a prompt SB in DNA. Later, C-N and C-O bond cleavage was found to be greatly reduced next to an abasic site, especially with LEEs of 6 eV compared to those at higher energy [41]. This suggested that the base moiety serves as an antenna for the initial capture of LEEs. The ability of each base to undergo base release (C-N bond cleavage) or SBs (C-O bond cleavage) was examined in trinucleotides with different central nucleotides flanked by thymine bases [126]. These studies showed that, for 10 eV electrons, the total damage decreased with the electronegativity of the DNA base (T > C > A > G); however, the amount of SBs was not significantly different between the four bases. Other base modifications, including 5,6-dihydrothyminee and 5-hydroxymethyluracil products, were identified in additional similar studies [123,128]. The formation of 5,6-dihydrothymine likely arises from decay of the corresponding TA to a ground state radical anion. The radical anion of pyrimidines is well known to undergo protonation and produce stable 5,6-dihydropyrimidine derivatives once dissolved in aqueous solution. In contrast, the formation of 5-hydroxymethyluracil was rationalized by C-H bond cleavage from the methyl position of thymidine. Lastly, Madugundu et al. and Khorsandgolchin et al. identified a novel class of DNA modifications arising specifically from the reaction of LEEs that could be used as biomarkers of DEA reactions [125,130]. These products represent the fragments that accompany those observed earlier, i.e., fragments with a terminal phosphate. The formation of this pair of products with a terminal phosphate and dideoxynucleosides can be explained by C-O bond cleavage by DEA, causing negative charge transfer to the oxygen of the phosphate moiety, while the radical resides on the C3′ or C5′ of the deoxyribose moiety. Indeed, this reaction appears to take place with very low activation energy, which is driven by the large electron affinity of the departing phosphate radical and the strong electronegativity of the phosphate group [50]. Because the yield of phosphate termini was 2-fold higher than dideoxyribose products, there are likely additional reactions occurring at the deoxyribose moiety that led to the formation of fragments with a terminal phosphate. The ensemble of the results from these detailed chemical analyses indicates three predominant types of LEE-induced products: C-N bond cleavage giving base release, C-O bond cleavage resulting in termini phosphate accompanied with the dideoxyribose product, and products with a 5,6-saturated double bond, i.e., pyrimidines and 5,6-dihydropyrimidines.

Chemical analysis reveals certain particularities about LEE reactions with DNA. Although all three types of damage form with both very low energy electrons (˂4 eV) and higher energy LEEs (6–15 eV), one can expect that their distribution changes with energy by proceeding through alternative pathways. For example, 5-hydroxymethyluracil that likely arises by DEA at the C-H of a base moiety was formed with 10 eV, but not with 1.8 eV LEEs [128,130]. The formation of dideoxyribose products, which predominates upon exposure to ~1.8 eV electrons, suggests that these products or analogous products from C3′ and C5′-centered radicals will be present at sites of LEE-induced SBs. Future studies in this direction will help determine the extent of these three pathways as a function of energy, level of hydration, and their formation in cellular DNA.

Another method was developed by the group of Solomun to achieve sufficient sensitivity for detecting the overall damage induced by LEEs to oligonucleotides [132]. Single DNA strands were tethered to a gold surface and exposed in vacuum to electrons with energies below that of the ionization threshold. The damage produced was analyzed ex vacuo by measuring the ability of the bombarded strand to hybridize with the dye-marked complementary strand via fluorescence and infrared (IR) spectroscopy methods. The damage was identified as SBs and found to correlate linearly with the number of guanines in the sequence, indicating enhancement of LEE-induced lesions by these bases [133]. These experiments have clearly shown that electron capture by the DNA bases, and particularly guanine, play an important role in radiation damage. Later, these authors found that, to immobilize DNA on glass, difunctional alkoxysilanes—instead of widely used trifunctional silanes—was more favorable for DNA microarray applications [134]. The method was further applied to investigate the binding of DNA to proteins. They investigated the interaction of a gene-5-protein with single-stranded DNA oligonucleotides (dT**_25_**) tethered to a gold substrate by surface plasmon resonance and confocal fluorescence. A highly stable g5p–ssDNA complex was rapidly formed on the gold surface and involved cooperative protein–protein interactions within the binding to DNA [135].

In experiments with double-stranded DNA (e.g., plasmids), conformational modifications can be detected and quantified. Multilayer films are prepared by lyophilization [10,15,17,48] or self-assembly [136]. In the latter process, diaminopropane intercalation between the plasmids produces highly uniform films physisorbed onto a highly oriented pyrolytic graphite surface [136]. When the condensed multilayer films are bombarded in UHV, LEEs of energies varying from 0.5 to 100 eV can be produced by an electron gun having beam resolution of about 300 meV [48]. The electron energy is varied by changing the potential between the substrate (ground) and the center of the filament of the LEE source. The absolute electron energy is determined by measuring the lowest potential for electron transmission through the film, which is considered to correspond to zero eV. In most apparatus, the diameter of the electron beam and the working distances from the target can be varied between 2 and 50 mm and 10 to 50 mm, respectively.

**Figure 2:**
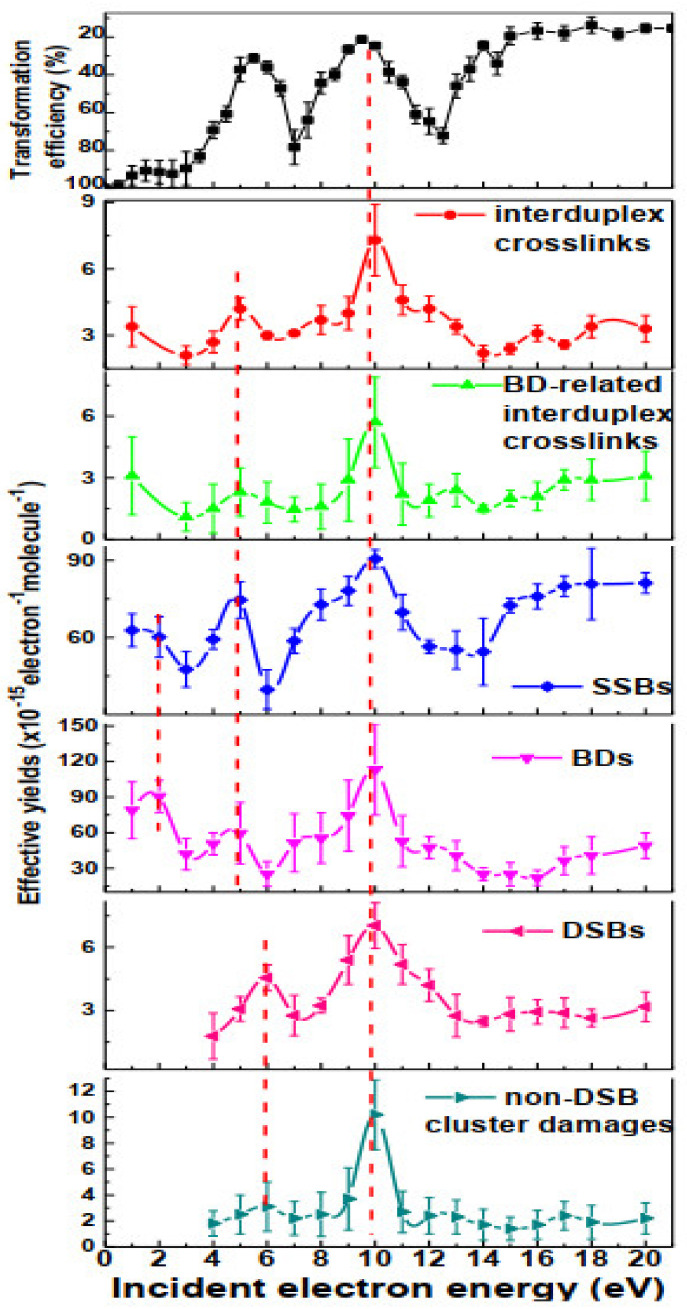
The upper frame is the complement of the transformation efficiency of JM109 E. coli cells, containing PGEM3Zf(−) plasmids bombarded with LEEs of 0.5 to 21 eV [137]. It is compared to the single-electron yields of interduplex and BD-related interduplex crosslinks, SSBs, BDs, DSBs, and non-DSB clustered damage, recorded as a function of electron energy. The dotted lines indicate the energies of TAs at 2, 5, 6, and 10 eV.

The different DNA conformations resulting from bombardment are quantified by imaging the respective bands in an electrophoresis gel; these bands can be related to SSBs, DSBs and inter-duplex CLs. After treatment of the bombarded samples with enzymes, enhancements of conformal damages can be identified as due to isolated BD, non-DSB clustered damage, and CLs related to BD [15]. To quantify the damage to plasmid DNA, it is also possible to directly measure the transformation of bacteria. The latter are incubated in an antibiotic-rich environment that would normally destroy them [137]. However, if plasmid DNA that encodes an enzyme capable of inactivating the antibiotic is artificially transferred into these cells, they can survive [138,139]. Should the plasmid DNA be exposed to LEEs before being transferred, then cell resistance against the antibiotic is reduced due to the imparted damage, and the transformation efficiency of the bacteria is diminished. The complement of the transformation efficiency (or survival) of *E. coli* bacteria on the energy of electrons damaging the injected plasmid is shown on the top of Figure 2.

Folkard et al. conducted the first measurements of yield functions for SSBs and DSBs induced by electrons in plasmid DNA films [140]. From the results recorded within the energy range 25 to 4000 eV, they suggested that the threshold of SSBs could be lower than 25 eV and that of DSBs lied between 25 and 50 eV [140]. Later, improvements in the technique made it possible to measure such yield functions with much lower energy electrons [12]. The yield functions of BDs, SSBs, CLs (including BD related CLs), DSBs, and non-DSB clustered lesions induced by 1−20 eV electron impact on five-ML plasmid films are shown in Figure 2. The maxima at 5 and 10 eV are caused by core-excited TAs decaying into bond-breaking channels. Two other peaks from similar resonances can be seen at 6 and 10 eV, in the DSB and non-DSB clustered damage yield functions. The increase at 2 eV in the SSB, BD, and CL yield functions is due to the formation of a shape resonance, previously identified at 2.2 eV by Martin et al., as a temporary attachment of an electron to a base followed by electron transfer to the phosphate group [49]. Electrons with energy E<4 eV cannot induce clustered damages to DNA. As shown in Scheme 1, it requires more energy to form an electron-binding electronically excited state of a base. The resonance peaks in clustered damage (i.e., DSB and non-DSB) yield functions lie at about the same energy as those of the complement of the transformation efficiency or cell survival shown on top of Figure 2 [137], indicating that not only clustered DNA damages induced by LEEs cause cell death, but that most of these potentially lethal damages arise from the decay of core-excited TAs. *Such a correlation demonstrates that clustered damages induced by LEEs are cytotoxic* [15]. A unifying mechanism for all DNA damages was proposed [15,141], from which Scheme 1 is derived.

Similar experiments were performed by the group of Orlando, with films of partially hydrated P14 supercoiled DNA plasmids of 6360 base pairs, in the range 5–25 eV [142]. Their results are similar to those reported in Figure 2, but with maxima in the yield functions of SSBs and DSBs shifted to higher energies by at least 2 eV. This difference was explained theoretically by electron diffraction, which depended on the base-pair sequence, and the formation of H_2_O-DNA complexes [142]. This latter hypothesis corroborated that advanced by Ptasinska and Sanche to explain their results on ESD from hydrated GCAT films [90].

As explained in the previous subsection, the photoelectric effect can also serve as a LEE source. Cai et al. employed photoemission from a tantalum foil, irradiated with 1.5 keV Al_Kα_ X-rays in UHV [143], to produce LEEs that reacted with a PGEM-3Zf(-) plasmid DNA in monolayer and thick (20µm) films deposited on the foil. By changing the thickness of the film, the damage caused by SEs from the metal substrate could be compared to that induced by the X-rays. The conformational damages were measured ex-vacuo by electrophoresis. The secondary LEEs had an average energy of 5.8 eV and a distribution peaking at 1.4 eV. The G values (i.e., yields per energy deposited in DNA) for SSBs and DSBs induced by LEEs were 86 ± 2 and 8 ± 2 nmol/J, respectively, which was 1.5 and 1.6 times larger than those induced by 1.5 keV photons [143].

## 5. LEE-Biomolecular Films Experiments at Atmospheric Pressure

Cai et al. were first to investigate LEE-induced DNA damage at atmospheric pressure [144]. Thick and thin films of pGEMt-3Zf(-) plasmid DNA were deposited on a tantalum foil and exposed to soft 15-keV (effective energy) X rays for various times under a relative humidity of 45% and 84% (Г = 6, Г = 21, expressed as moles of water per mole of nucleotides) in the laboratory atmosphere. X-ray-induced SE emission from tantalum resulted in a significant enhancement factor (EF) relative to vacuum for SSBs, DSBs, and CLs with the thin films, i.e., EFs of 3.8 ± 0.5, 2.9 ± 0.7 and 7 ± 3 at Г = 6 and 6.0 ± 0.8, 7 ± 1 and 3.9 ± 0.9 at Г= 21, respectively. The study illustrated that the hydration level of DNA influences the DNA damage EFs with LEE emission from the thin foils [144]. Similar results were previously obtained with gamma radiation [145,146].

The initial investigations of Cai et al. opened an area worthy of further research, which led to the construction of an apparatus to investigate the yields of LEE-induced biological damage under controlled environments at SATP. In this new system, which allowed moving closer to cellular conditions, the biomolecular films were deposited on clean metal substrates surrounded by a pure gas or vapor (or a mixture thereof) [147,148,149,150]. Photo-emitted 0–30 eV electrons were produced with 1.5 keV Al_Kα_ X-rays incident on the metal substrate. Most of the results were recorded with a tantalum substrate, giving a photoelectron distribution with a mean energy of 5.8 eV and modal energy of 1.4 eV. The differences in the damage yields obtained with the metal and glass substrates was attributed to the interaction of the photoelectrons with the deposited films. Results obtained from irradiation of 5 ML films of plasmid DNA with this improved method are listed in Table 1 for different N_2_, O_2,_ and N_2_O atmospheres and humidity (Г). In these environments, the gases are expected to reside onto the DNA molecule for a period that depends on gas-solid equilibrium at SATP, whereas water layers of different thicknesses form on the DNA depending on the humidity level [149]. Thus, the results reported in the following paragraphs must be interpreted as resulting from two sources: perturbation of the DNA by the presence of the gas and/or water at its surface and the indirect effect of LEEs [150], producing highly reactive radicals by their interaction with the adsorbed molecules or the water layers.

Brun et al. first compared the yields of the loss of the supercoiled configuration of plasmids irradiated under vacuum and a humidity of 65% of the laboratory atmosphere [147]. G values for total DNA damage induced by LEEs were determined to be 400 ± 200 and 600 ± 200 nmol/J, respectively [147]. Later, Alizadeh et al. obtained G values of 227 ± 15 and 415 ± 15 nmol/J with more precision for the same damage under pure dry nitrogen or oxygen at SATP, respectively [148]. When changing the atmosphere from N_2_ to O_2_, this damage increased by factors of 1.9 and 1.8 for X-rays and LEEs, respectively. The results indicate two possible mechanisms related to the oxygen effect: (1) the radicals and ions produced by the interaction of LEEs with O_2_ are more reactive than those arising from N_2_ [148,151] and (2) the presence of O_2_ enhances the efficiency of DNA degradation pathways by further interacting with the induced reactive radicals. The latter hypothesis is more compatible with the ESD investigations [91,92]. Moreover, oxygen reacts with carbon-centered radicals that are produced by the reaction of X-rays and LEEs with DNA. In this way, the damage is fixed, meaning that you block other pathways (radical-radical; radical plus electron), which can reconstitute the target molecule. These results were later confirmed with dry DNA film (Γ = 2.5) irradiated in 100% oxygen, providing LEE G values for total damage and SSBs of 473 ± 123 and 432 ± 112 nmol/J, which were 1.8 and 1.7 times higher than those of the dry film in nitrogen [148]. Under 100% humidity (Γ = 33) in a nitrogen atmosphere, the G values of total damage, SSB, and DSB were 1852 ± 482, 1545 ± 403, 21 ± 5 nmol/J, respectively. The former two are 7.1 and 6.2 times the G values under nitrogen at Γ = 2.5 [149]. When a nitrogen atmosphere of 100% humidity (Γ = 33) was changed to an O_2_ environment of the same humidity level, these G values increased by factors of 3.1, 3.6, and 2.2, respectively [151]. The results show that, contrary to the scavenging effect of O_2_ on e_aq_, LEE interaction with DNA is significantly enhanced by O_2_ in an atmosphere saturated with water.

More recently, Hahn et al. measured damages induced to herring sperm DNA by X-ray photons and the generated secondary LEEs under UHV, as well as in atmospheres of N_2_ and water molecules [154]. The latter provided 5–10 water molecules per nucleotides. From monitoring the magnitude of the modification of specific bonds and comparison of the results under the different environments, they were able to distinguish between the direct and indirect effect of HER [154]. A strong increase was found in total DNA damage upon hydration. Dry DNA showed a propensity for the induction of strand breaks, whereas 2-deoxyribose and nucleobases were affected significantly less by direct damage. In the presence of water, the excited water molecules and hydroxyl radicals modified the damage distribution, leading to a strong increase in base damage and release.

Alizadeh et al. further investigated the damage to DNA film induced by LEEs under nitrous oxide (N_2_O), a potential radiosensitizer [152]. In this case the G-values for loss of supercoiled DNA and SSB (i.e., 737 ± 110, 540 ± 80 nmol/J) were increased by 1.6 and 1.3 relative to those obtained under oxygen. Cell radiosensitization by N_2_O may therefore involve reactions between LEEs and N_2_O molecules near DNA. A similar experiment was designed, in which 0–1.5 eV LEEs were generated by UV light incident on tantalum or n-Si substrates instead of X-rays [153]. The G-value of SSBs induced by 0–1.5 eV LEEs under a dry nitrogen atmosphere was 47 ± 37 nmol/J [153]. This later value can be compared with those in Table 1 obtained with X-rays under dry nitrogen. The comparison indicates that the TAs formed below 1.5 eV are about 5 times less effective in causing SSBs than electron interactions resulting from the 0–30 eV X-ray photoelectron distribution escaping the tantalum substrate.

## 6. LEE Experiments with Clusters

### 6.1. LEE Impact on Clusters Containing DNA Constituents or Their Analogs

The electron scattering and attachment experiments with gaseous isolated molecules, where no influence of any environment exists, cannot represent adequately the action of LEEs in cells. Cluster-phase studies can be considered as a bridge to connect the gas and condensed phases to transfer basic knowledge from simple systems to more complex conditions, as presented in Section 3 [37,50,57]. One problem with large biomolecules is that they are often easily decomposed when vaporized (i.e., heated) for inclusion into a cluster of atoms or molecules [155]. Despite this difficulty, Förstel et al. [156] produced a molecular jet of microhydrated adenine and uracil without significant decomposition of these biomolecules as shown by MS analysis. In a similar experiment, Kočišek et al. [157] measured the fragmentation patterns arising from DEA to microhydrated dCMP. In another type of experiment, the vapor from a heated sample of biomolecules was seeded into a helium jet [158]. Uracil and derivatives were found to remain stable up to maximum temperatures of 300℃, indicating that further LEE scattering or spectroscopic studies could be performed on the intact compounds [158]. The experiments with DNA-related biomolecular clusters were performed with a crossed electron–molecular beam coupled to a MS.

Cluster environments can strongly influence the DEA process, where the “caging” effect can return the dissociating fragments toward their initial positions, thus increasing vibrational energy transfer to the surrounding medium. Furthermore, the TA spends more time with internuclear distances of potential fragments in the autoionization region, thus increasing the electron autodetachment probability. Both phenomena can reduce bond scission. In large biomolecule fragmentation induced by LEEs, “caging” was first investigated with hydrated peptides [159] and in clusters of the amino acid derivative betaine [160]. The stability of cluster cations formed by the assembly of tryptophan or serine molecules inside charged helium droplets and subsequent droplet evaporation was later investigated by Tiefenthaler et al. [161]. Neustetter et al. were first to provide a detailed experimental investigation of the effect of solvation, on TA formation and decay, in pure and hydrated clusters of a DNA and RNA subunit prototype [162]. Their target was the nucleobase prototype pyrimidine [162]. In contrast to the results obtained with the isolated or hydrated molecule, these authors observed the molecular anion in pure pyrimidine clusters. However, solvation prevented ring breakage of pyrimidine after electron capture, as well as fragmentation of other bonds. Hindrance of uracil and thymine fragmentation induced by 0–3.5 eV electrons in microhydrated clusters was later demonstrated by Kočišek et al. [163]. They suggested that such observations could be attributed to the “caging” effect, including fast energy transfer to the solvent, occurring essentially via motion of the H fragments. This process can redistribute the internal energy of the target, as the vibrational energy of H atoms in the target molecule couples with the vibrational modes of the surrounding water molecules. This vibrational quenching can lead to stabilization of base anions in water clusters [163]. Not only does it depend on the presence of water molecules, but it could be influenced by specific hydrogen-bonding sites and on the number of H_2_O molecules at that site, as seen in enhanced fluorescence of 2-aminopurine in solution [163]. Modification of the lifetime and energies of the TAs of the bases can also be modified by the presence of water, thus changing the magnitude of the decay channels.

From the fragmentation patterns of 0.5–13 eV electron attachment to dCMP, the microhydrated deoxynucleotide was found to decompose via DEA causing primarily dissociation of C-N glycosidic bond and to a lesser extent scission of the P-O bond [157]. However, the magnitude of these SBs was reduced by hydration and their relative contributions modified. Similar experiments with 5-nitro-2,4 dichloropyrimidine were compared with those on the isolated compound in the gas phase; they indicated a strong bond specificity for DEA reactions, which could be related to the role of LEEs in the synergism of chemoradiation therapy (CRT) [164]. Experiments with neutral clusters, including DEA suppression and enhancement due to cluster environments have recently been reviewed with emphasis on microhydration [165].

### 6.2. LEE Photodetachment from Anions in Binary and Larger Clusters

Instead of an external source of LEEs impinging on a target of condensed DNA constituents, it is also possible to generate LEEs from inside condensed matter with incident external photons. Thus, LEE-molecule interactions with condensed biomolecules can be investigated by photodetaching an electron from a stable anion surrounded by biomolecules forming a cluster or from an anion electrostatically bound to a biomolecule. The iodide anion has often been used in such studies as a source of photoelectrons of specific energy [166,167,168,169,170,171,172,173]. In such experiments the detaching LEE can be captured by a molecule within the cluster to form a TA. The subsequent dynamics can be probed by analysis of photofragments and time-resolved photoelectron spectroscopy [166,167,168,169,170,171,172,173].

The photodissociation dynamics of the binary anion cluster iodine-uracil (I^−^·U) [173] revealed the production of I^−^ ion photofragments and smaller yields of dehydrogenated anion nucleobases (i.e., [U–H]^−^). The fragment ion yields displayed two peaks at photon energy ∼4.0 and ∼4.8 eV, assigned to excitation of a dipole-bound excited state of the complex and excitation of a uracil-localized π-π* transition, respectively. These studies were proceeded by time-resolved photoelectron spectroscopy measurements, capable of following the dynamics of electron attachment and autodetachment of electrons from iodine anions and photodissociation from iodide embedded in clusters of fundamental DNA constituents [174,175]. The time-resolved dynamics of electron attachment and transient decay in the nucleobases [169], uracil [170,171,173], thymine [170,172], and adenine [176], including uracil–water clusters (i.e., I^-.^U^.^H_2_O) [177] were of particular interest. In those studies, the group of Neumark was able to observe the formation, evolution, and decay of both dipole-bound and valence-bound anions from the nucleobases in these clusters. The presence of water was expected to stabilize the valence-bound and suppress dipole-bound anions, indicating new prospects for the application of the technique in further studies of the microhydration kinetics of nucleobases, as well as electron attachment and photodissociation of more complex nucleic acid components [174,176,178].

Prior to these experiments, Osterwalder et al. had proposed a high-resolution anion photolysis spectroscopy technique that combines velocity map imaging and anion threshold photolysis [179]. Parsons et al. used the velocity mapping imaging with photodissociation energy of 3.496 eV to generate anion photoelectron spectra of deprotonated thymine and cytosine [180]. In other ultrafast photoelectron spectroscopy investigations, the energy required to eject hydrated electrons from a very large amount of clustered water molecules (i.e., a liquid jet) into a vacuum without changing the configuration of the solvent was measured [181,182]. Solvated electrons bound at the water surface and solvated electrons in the bulk solution had vertical binding energies of 1.6 eV and 3.3 eV, respectively, with lifetimes longer than 100 ps. Slow electron velocity imaging of negative ions its applications in spectroscopy and kinetics as well as the dynamics of electron solvation in molecular clusters [183] has been summarized by Neumark [182,184].

## 7. LEE Reactions in Solution

### 7.1. LEE Reactions Investigated by Pulse Radiolysis

The formation of TAs in solution has recently been observed by pulse radiolysis [185]. From transient spectroscopic analysis, quasi-free electrons with energy near the conduction band of liquid diethylene glycol were found to effectively attach to ribothymidine leading to a new absorbing species characterized in the UV-visible wavelength region [185]. This absorption decayed with a time constant of ~350 ps, exhibiting little change in ribothymidine concentration. Based on the time-resolved data and theoretical calculations, the intermediate species was assigned as an excited anion radical, undergoing N1-C1′ glycosidic bond dissociation via DEA rather than autoionization or relaxation to its ground state [186]. Pre-solvated electrons were not involved in the process. Reactions of these latter species with fundamental DNA constituents were investigated in water solutions by femtosecond-laser photoelectron-detachment spectroscopy [187,188,189] and recently by pulse radiolysis [190,191,192]. Relevant information on reactivity of LEEs with hydrated ribothymidine was recently obtained by picosecond electron pulse radiolysis associated with femtosecond pump-probe laser spectroscopy [190]. The ensemble of the results of Ma et al. has recently been reviewed in detail and explained in the context of radiation chemistry [186].

### 7.2. Secondary LEEs Generated by a Membrane Irradiated by Fast Electrons

The electron irradiation of immobilized DNA in a water solution was conducted by Solomun and co-workers [193]. High-energy electrons passed through a 40-nm thin SiO_2_ membrane on which duplex DNA was anchored. DNA was labeled with a dye and its damage by irradiation could be detected in situ by fluorescence. Corresponding MC simulations showed that the spatial distribution of ionizing events in water could be controlled by the initial electron impact energy. Since the dynamics and reaction ranges of hydroxyl radicals (•OH) and LEEs are different, it was possible to tune experimental conditions to principally •OH or secondary-electron reactions [193]. In another similar experiment, a 30 keV electron beam from a scanning electron microscope traversed a 100-nm thick Si_3_N_4_ membrane to irradiate a small target of pUC19 plasmid DNA dissolved in water [194]. DNA SSBs and DSBs were measured by gel electrophoresis. MC simulations indicated that the kinetic energy spectrum in water consisted of 99% electrons of energies below 1 keV, with a distribution of 25% in the range 0–20 eV, 40% between 20–40 eV, and 10% between 40–60 eV. Most of the damage was attributed to the indirect effect of secondary species created by these electrons. The ratio of their G values of SSBs to DSBs is 14, which is much lower than that reported by other authors in humid or dry samples (e.g., see Table 1). These differences may be due to the different kinetic energy spectra of electrons, higher amount of surrounding water, and modification bond-breaking channels in solvated DNA. The authors noted that the technique, when combined with theoretical simulations, could be applied to quantitatively measure radiation damage to DNA in solution by electrons under different conditions (pH, salinity, co-solute) [194]. Adding GEANT4 particle-scattering simulations in water and calculations concerning the movement of the biomolecules, led to the development of a general method to determine energy deposits in a biologically relevant target volume of dissolved biomolecules irrespective of the experimental setups [195]. The calculation showed that fewer than two ionizations could produce a SSB with median energy of about 6 eV, which is much lower than 17.5 eV, i.e., the energy for SB induction by ionization applied in conventional theoretical models [196,197].

Much of the research of Solomun and co-workers using this method has been concentrated on the action of ectoine on irradiated DNA. Ectoine is a small molecule that, aside from being an •OH scavenger, stabilizes proteins and other cellular structures [198,199,200]. It is a compatible solute and osmolyte, known to be an effective protectant of biomolecules and whole cells against heating, freezing, and extreme salinity [201]. They irradiated plasmid DNA pUC19 in aqueous solution at various ectoine and NaCl concentrations. The presence of ectoine reduced DNA damage due to a decrease of SE production near the molecule, caused by displacement of water in the extended hydration shell [199]. The local hydration shell around ectoine and its influence on the binding of a gene-5-protein (G5P) to a single-stranded DNA (dT25) also protected DNA against SE damage [202].

### 7.3. Femtosecond-Laser Induced Cold Low-Density Plasmas

Compared to HER, only electrons of low energies are produced in solutions, along with parent cations (or holes) using techniques of femtosecond laser filamentation [203,204,205], as well as IR, visible and UV light incident on various types of plasmonic nanostructures [206,207,208,209,210]. Within intense ultra-short femtosecond laser pulses in a solution, self-focusing of the electromagnetic radiation produces a strong electric field leading to local multiple ionizations and hence a plasma initially containing cations with LEEs, whose energy lies between zero and a cut-off value. The latter was calculated by Liang et al. from a multi-rate equation model [205]. It turns out that in water, these LEEs have only small energies, on average below 0.5 eV at short laser wavelengths (515 nm), but reach up to 14 eV, depending on experimental conditions, at longer laser IR wavelengths (1030 nm). Thus, cold low-density plasma generation can serve as a pure source of initial cations and electrons of energy lower than 15 eV. Furthermore, the long laser IR wavelengths provide a suitable therapeutic window for radiation penetration into biological tissue.

Using a spatial phase shaping technique, the filamentation of femtosecond laser pulses could be optimized in an ethanol solution containing a fluorescent dye [211]. The generation of laser-induced low electron-density plasma channels could also be controlled in DNA aqueous solutions, so as to minimize the unwanted thermo-mechanical effects associated with plasma of higher density [211]. The laser beam was observed to penetrate about one cm in water. This irradiation method was shown to emulate an adjustable high-dose rate ionizing radiation source by studying the femtosecond laser-induced synthesis of gold nanoparticles (GNPs) in gold chloride aqueous solutions [212,213].

Belmouaddine et al. measured the damage induced to DNA bases dissolved in water by this laser technique in the absence and the presence of oxygen [204]. The number of DNA base modifications per energy deposited was compared to that induced by UV and γ-rays [204]. As shown by the analyses of photo-induced, oxidative, and reductive DNA base products, the effects of the cold plasma were mainly mediated by reactive radical species produced by water ionization, rather than by the strong laser field. Reactions between the primary radicals within the plasma resulted in a dramatic decrease in the yields of DNA damages relative to sparse ionizing radiation, such as γ-rays. The intense radical production also depleted locally the oxygen concentration. The results were almost the same as to those of the radiolysis of water induced by high LET particles. Thus, as expected, biomolecular damage in water triggered only by initial cations and LEEs was very similar to that arising from HER.

Intense ultra-short IR laser pulses can deposit a very large dose up to 10^11^ Gy/s inside an adjustable and well-controlled macroscopic volume, without any energy deposition in front or behind this target volume [203]. The dose rates exceed by orders of magnitude those provided by the most intense clinical radiotherapy systems. The overall characteristics are not only similar to conventional high LET ionizing radiation, but as shown in an animal cancer model, this method can deposit a therapeutic radiation dose as deep as few centimeters into tissue with minimal energy deposition along the penetration length [203]. From such experiments in mice, a new type of cancer laser treatment based on such high-power IR pulses was proposed [214] and evaluated in vivo [203]. Finally, we note that LEEs could also play an important role in the laser-produced low-density plasmas used in micro- and nano-surgery [215,216,217,218,219,220].

## 8. Applications to Radiation, Chemoradiation, Targeted-Radionuclide, Gold-Nanoparticle and Laser Therapy

The investigation of the interaction of LEEs with DNA and its fundamental constituents has implications for radiotherapy in combination or not with chemotherapy, as well in estimating health risks from environmental HER on earth and in space [197,221,222,223]. Quantification of LEE-induced DNA damages for determination of the radiobiological effectiveness of HER is particularly needed in the development of new radiotherapeutic modalities, including heavy-ion radiotherapy, targeted radionuclide therapy and nanoparticle-aided radiotherapy. In such applications, the CSs for LEE-induced DNA damages become important input parameters in Monte Carlo (MC) simulations to generate microscopic absorption doses and quantify, down to the nanoscopic level, the biological effects of HER [224]; e.g., in targeted radiation therapy, where short-range radiation emitted from a suitable radionuclide is absorbed within sub-micrometer ranges. In doing so, these initial particles produce locally enormous quantities of LEEs, whose interaction CSs are needed for dose calculations. Recently, methods have been developed to obtain absolute CSs for LEE-induced DNA damages from thin-film experiments, by applying mathematical survival models to the raw data [225]. CSs for total DNA damage, CLs, SSBs, and DSBs have been reviewed over the 0.1 to 100 eV range [75]. All CSs determined by electron impact on thin DNA film are considered lower-limit values due to experimental factors. In fact, according to the results of Alizadeh et al. [148,149], it is quite realistic to conjecture that the CSs derived from dry-DNA films in vacuum could be increased by an order of magnitude, when LEE-interaction with DNA occur at atmospheric pressure in an oxygenated and hydrated environment, where the indirect effect of LEEs must be considered.

As first demonstrated by Zheng et al. [226], binding a platinum (Pt)-based chemotherapeutic agent (CA) to DNA increases LEE-induced damage to the molecule, indicating that LEEs, produced in large number by HER, are implicated in cell radiosensitization by such CAs and hence in concomitant CRT. The role of LEEs in concomitant CRT was more recently verified in cells [227] and tumor-bearing mice by Tippayamontri et al. [228,229], and in rats by Charest et al. [230], with the Pt-based CAs cisplatin, oxaliplatin, and carboplatin, which are among the most commonly used anti-cancer drugs in the clinic [231]. An EF larger than unity, defined as the ratio of the measured damage in the Pt-CA-DNA complex divided by that in non-modified DNA was consistently observed in previous studies upon LEE irradiation [215,232,233,234,235]. Absolute CSs have also been measured for LEE-induced damage to CA-DNA complexes. For example, when the CA cisplatin is chemically bound to DNA in a ratio of 5:1, the CSs of potentially lethal DSBs and non-DSB clustered damages at 10 eV increases to 9.3 ± 0.4 and 8.2 ± 0.3 × 10^−15^ cm^2^ per nucleotide, respectively; i.e., it increases by factors of 2.2 and 1.3 compared to unmodified DNA [236]. Other CS values for induced LEE damage to CA-DNA complexes have recently appeared in the literature [75,197]. All results show that the increase in DNA damage is large enough to justify detailed MC simulation, which could provide a quantitative assessment of DNA sensitization by Pt drugs (i.e., CAs). MC calculations may also help design new more effective CAs for CRT [224]. More information on the role of LEEs in CRT is available in recent review articles [222,237,238].

Other compounds acting only as radiosensitizers also involve LEE interactions [39]. For example, a halogenated base can replace a natural base during cellular DNA synthesis [239]. In this case, radiosensitization occurs via efficient DEA to these halogenated DNA molecules [240]. However, hepatic dehalogenation by thymidylate synthetase prevents achieving therapeutic levels of halouridines in cancer cells in vivo [239]. Despite their high potential as radiosensitizers for cancer treatment by radiotherapy, these drugs are therefore not applied in the clinic. However, now that we know and understand the basic radiosensitizing mechanisms of these compounds [39], it becomes possible to design new molecules [241,242,243,244,245] that act like halouridines, with similar or even better DEA efficiency, but probably without their in vivo drawbacks.

It has been amply demonstrated that when irradiated living cells containing GNPs, their survival can be considerably reduced compared to those which do not [246]. Particularly, when under irradiation with relatively low energy photons (20–100 keV), GNPs produce EFs that range from 1.14 to 20 in the decrease of cancer cell survival [246]. The role of LEEs in the enhanced killing of cancer cells containing GNPs has been reviewed in recent articles and book chapters [237,247,248]. As indicated in picosecond pulse radiolysis experiments, the very fast reactions of electrons with water provides an enhanced oxidizing zone around GNPs [249], which could also be related to their radiosensitizing behavior in cells.

GNPs enhance the local production of LEEs [248] and Pt-CAs render DNA more sensitive to LEEs [222]. From this knowledge, it has been suggested that if Pt-CAs are bound to DNA and the GNP are either bound to the molecule or localized within 10 nm, the EF for DNA damage would increase beyond the values around 2.3 for LEE-induced SSBs and DSBs found for these agents bound individually to DNA in ratios of 1 or 2 per molecule [250]. With similar ratios of both agents bound to DNA, irradiated with 60 keV electrons, Zheng et al. found that the EF of DSBs increased by a factor close to 4 [250]. This finding prompted the development of liposomes capable of carrying Pt-CAs preferentially into cancer cells [230,251,252]. Recently, Charest et al. succeeded in fabricating similar liposomes containing both Pt-CAs and GNPs [253]. Progression of HCT116 tumor implanted subcutaneously in NU/NU mice was monitored after an irradiation of 10 Gy combined with either GNPs, carboplatin, or a liposome containing both, injected directly into the tumor. Radiosensitization by GNPs or carboplatin alone was not observed at low concentration of the separated agents. However, the same low concentrations of carboplatin and GNPs administered simultaneously by encapsulation in liposomal nanocarriers led to efficient radiosensitization and control of cell proliferation [253].

Aside from their major role in radiotherapy, LEEs are also strongly implicated in the processing of biological materials by femtosecond lasers [203,215,219]. Such new techniques can follow, in real time, the subsequent generation of pre-solvated and solvated electrons and their reactions [216,217,218,254]. Besides these latter species and reactive radicals, IR laser-induced plasmas produce high-density avalanches of 0–14 eV electrons in solution [204,205]. These electrons are suspected to be involved in the destruction of cells, via DNA bond breaking, in ultrafast-laser nano-surgery, and laser cancer therapies [215,216,217,218,219,220].

## 9. Conclusions and Future Challenges

Measuring the survival of cells irradiated by HER with respect to a few parameters, such as intensity and type of radiation, is rather straightforward. However, understanding what happens in a cell as a function of time from the very first interaction of HER with the atoms of the cell’s biomolecules to the chemical reactions and cell response, leading to its final condition, presents an enormous challenge. The complexity of cellular media makes it difficult to extract the details involved and hence different models have evolved to reduce the number of parameters and possible interactions. Such models can be divided into two major categories, i.e., those representing the direct and indirect effects of HER. The latter model often consists of a biomolecule in a water solution, where the reactions of radicals and other radiolysis products with the solute can be investigated. In addition, water solutions are relevant systems for investigating the production of cations and their reactions with DNA bases, as they may occur in cells. In fact, it has been shown that the generation of pyrimidine and purine base cations in aqueous solutions of isolated DNA upon exposure to high intensity 266-nm nanosecond laser pulses mimics well the ionization of the bases, which is part of the direct effect of HER [255]. In these studies, the link to the action of HER on cellular DNA was made in terms of distribution of oxidation products. It may be added that oxidation reactions triggered by •OH, which are the main reactive species generated by the indirect effect of ionizing radiation, give rise to similar DNA base degradation profiles in aqueous solutions and cells [256,257,258,259]. Such results support the suitability of aqueous systems to provide conditions for assessing the indirect effects of ionizing radiation on cellular DNA.

In the other model, a biomolecule (e.g., DNA) can be irradiated at atmospheric pressure or in vacuum, with or without being surrounded with other molecules. The experiments can be conducted at room temperature or at much lower temperature to immobilize the reaction products [24,40]. The latter can be analyzed in situ or outside the irradiation environment. In both the dry state and in water, efforts are made to understand the time dependence of the sequence of events leading to specific damages. However, these models are far from representing the complexity of the cellular medium. This complexity should be considered, for the next decades, as the major challenge to our understanding of the succession of events in radiobiology, i.e., compiling and understanding the present and future results obtained with these model systems and developing intermediate and more complex models to obtain a more complete picture of the effect of HER in cells.

As seen in this review, our comprehension of the direct effect of radiation on DNA has progressed enormously during the last two decades, with respect to our knowledge of LEE interactions. We now know that LEEs interact strongly with DNA via the formation of TAs and attach usually first to the DNA bases, with a preference for guanine in the range 0–4 eV. These TAs can decay by autoionization or DEA. The latter process can damage dry DNA over the entire 0 to 12 eV range, whereas autoionization can be destructive above the electronic excitation energy threshold. However, these decay channels are influenced by other relevant biomolecules surrounding DNA. According to theory and cluster experiments, below about 4 eV, the damage to small DNA constituents (e.g., the bases and nucleotides) is considerably suppressed, when they are surrounded by water molecules. This decrease in DEA has been attributed to a perturbation of the lifetime and change in the energy of the shape resonances below 4 eV. In particular, the upward energy shift of the curve-crossing of the extra-electron π and σ orbitals of the base and phosphate groups, respectively, considerably reduces electron transfer from the bases to the phosphate group and thus decreases the number of SSBs. Above this energy, the presence of water around single and double DNA strands increases damage yields, as reported in this review. In long sequences of duplex DNA, bond scission occurs with or without intramolecular transfer of an electron detaching from a TA; such transfer usually occurs from a base to the phosphate group. *From these processes, it was shown that within femtoseconds after their creation by ionization, LEEs can induce potentially lethal DNA lesions.*

Many major challenges exist in this field. Ongoing investigations of LEE-biomolecule interactions in thin films, as well as in water and other solvents by secondary- or photo-electron emission from clusters, immersed surfaces or nanostructures, ultra-fast pulse radiolysis, and femtosecond laser-induced low-density plasmas are highly promising. Nevertheless, both thin-film and biomolecular-cluster experiments would benefit from the development of more sophisticated methods to evaporate large biomolecules isolated in vacuum (e.g., laser induced desorption). Furthermore, there are advances to be made even to explain theoretically the present experimental results. Even more challenging is the replication of the cellular environment under conditions enabling different parameters and reactions to be determined. Ultimately, we would like to directly probe the reactions of specific radiation-induced species within cells as a function of time.

As far as experimental investigations are concerned, it is difficult to imagine how one could measure products created by monoenergetic electrons injected with nanometer precision at a specific position in a cell, without perturbing its functions. Even more difficult would be to determine the reaction pathways. Thus, for the next decade, it is probably more realistic to assemble and generate knowledge from various biologically relevant experiments and calculations. More specifically, the main avenues for future developments could include: (1) refinement of thin film fabrication on appropriately chosen surfaces to construct multi-molecular cellular environments, (2) refinement of methods capable of investigating the reactions of LEEs with biomolecules in solution, (3) further development of cluster beam techniques to progressively embed in a medium (e.g., water) biomolecules of increasing complexity, (4) enhancement of quantum-mechanical theoretical and computational capabilities for treating more complex and larger LEE-biomolecule systems, so as to emulate more adequately biomolecular environments, (5) refinement of MC codes to increasingly include LEE scattering and attachment CSs and thus provide reasonable estimates of the local dose distributions and biological effectiveness at the level of the cell, nucleus, and DNA, (6) additional measurements of absolute LEE scattering CSs in the condensed phase, for incorporation as input parameters into MC codes, and (7) increased interdisciplinary research between the LEE community, biologists, and clinicians, to apply rapidly fundamental discoveries to radiobiology and radiotherapy. When performed in combination with a variety of chemotherapeutic drugs, radiosensitizers, or nanoparticles bound to DNA, such interdisciplinary research should help to determine the ensuing enhancement of chemical and biological damage, and thus more directly contribute to the development of efficient cancer treatments.

## Figures and Tables

**Table 1:** Comparison of G-values for SSBs (G_SSB_), DSBs (G_DSB_) and loss of supercoil (total damage, G_LS_) induced by UV and X-ray photoelectrons emanating from a metal substrate covered with 5 MLs of plasmid DNA.

References	Photon Energy (eV)	Average LEE Energy (eV)	G_LEE_ (× nmol/J)			Hydration Level ^a^	Environment
			G_SSB_	G_LS_	G_DSB_		
Brun et al. [147]	1486	4.0		400 ± 200		Г = 2.5	vacuum
	1486	4.0		600 ± 200		Г ≈ 10	Air
Alizadeh et al. [149,150]	1486	5.85	248 ± 65	260 ± 50		Г *=* 2.5	N_2_, SATP ^b^
	1486	5.85	226 ± 59	247 ± 64	-	Г *=* 5 ± 1	N_2_, SATP
	1486	5.85	223 ± 57	309 ± 80	-	Г *=* 10 ± 1	N_2_, SATP
	1486	5.85	268 ± 70	412 ± 107	21 ± 5	Г *=* 20 ± 1	N_2_, SATP
	1486	5.85	1545 ± 403	1852 ± 482	21 ± 5	Г *=* 33 ± 1	N_2_, SATP
Alizadeh et al. [148]	1486	5.85		227 ± 15		Г *=* 2.5	N_2_, SATP
				415 ± 15		Г *=* 2.5	O_2_, SATP
Alizadeh et al. [148,151]	1486	5.85	206 ± 54	288 ± 75	10 ± 3	Г = 2.5	N_2_ + O_2_, SATP
	1486	5.85	432 ± 112	473 ± 123	-	Г *=* 2.5	O_2_, SATP
Alizadeh et al. [152]	1486	5.85	540 ± 80	737 ± 110	46 ± 66	Г *=* 2.5	N_2_O, SATP
Liu et al. [153]	3.1–5.2 ^c^	0.75	47 ± 37	49 ± 38	-	Г = 2.5	N_2_, SATP

a: Г, moles of water per mole of nucleotides. b: standard atmospheric temperature and pressure. c: produces electrons with maximum energy of 1.5 eV, calculated from subtracting the work function of substrate from the photon energy.

## Abbreviations

A	Adenine
BD	base damage
C	Cytosine
CA	chemotherapeutic agent
CL	crosslink
CRT	chemoradiation therapy
CS	cross section
DEA	dissociative electron attachment
DNA	deoxyribonucleic acid
DSB	double strand break
EF	enhancement factor
ESD	electron stimulated desorption
G GNP	Guanine Gold nanoparticles
HER	high energy radiation
HPLC	high-performance liquid chromatography
IR	infrared
LEE	low-energy electron
LET	linear energy transfer
MC	Monte Carlo
MS	mass spectrometry
PNA	peptide nucleic acid
SAM	self-assembled monolayers
SATP	standard ambient temperature and pressure
SB	strand break
SE	secondary electron
SSB	single strand break
T	Thymine
TA	transient anion
U	Uracil
UHV	ultra-high vacuum
UV	ultraviolet
XPS	X-ray photoelectron spectroscopy
